# Combination Therapies in Advanced, Hormone Receptor–Positive Breast Cancer

**Published:** 2018-01-01

**Authors:** Emily Olson

**Affiliations:** Mayo Clinic Cancer Center, Rochester, Minnesota

## Abstract

About 17% of women with breast cancer have locally advanced or metastatic disease at the time of diagnosis, and 30% to 40% of women diagnosed with early-stage disease will eventually have recurrence. The majority of breast cancers express estrogen or progesterone receptors, and hormonal therapies (HTs) remain the treatment of choice for these cancers after the resection of primary tumors. In addition to their effectiveness, HTs often have fewer severe side effects compared with chemotherapies. As breast cancer recurs or progresses, however, it becomes less responsive to successive HT, and the duration of response decreases. Recent advances have identified specific combinations of HTs that can extend the duration of response in appropriate patients with advanced breast cancer. Furthermore, research into signaling pathways has led to the availability of targeted agents that improve efficacy and duration of response when used in combination with specific HTs. Despite their effectiveness and advantages, these combination therapies increase the burden of side effects and the care required for proper management. In addition, practitioners must educate patients about the increasing complexity regarding treatment decisions, and provide care as part of a patient-centered team that optimizes both the medical outcomes and quality of life of patients with breast cancer.

Each year in the United States, approximately 250,000 women are newly diagnosed with invasive breast cancer (Howlader et al., 2017). Approximately 17% of those women have locally advanced or metastatic disease at the time of diagnosis (Iqbal, Ginsburg, Rochon, Sun, & Narod, 2015), but many more women diagnosed with early-stage breast cancer will eventually experience recurrence. Among all women diagnosed with breast cancer, almost 90% remain alive after 5 years, but the average 5-year survival is only about 27% among women diagnosed with distant metastases (Howlader et al., 2017). Therefore, researchers continue to seek better treatment options for women with advanced breast cancer.

Roughly 70% to 80% of breast cancers contain receptors for the hormones estrogen and/or progesterone (Huang et al., 2005; Iqbal et al., 2015). When naturally occurring estrogen binds to the estrogen receptor (ER) in breast cancer cells, it can stimulate growth of the cells and is the major driver of tumor growth in a majority of breast cancers (Osborne & Schiff, 2011). Hormonal therapies (HTs) for breast cancer aim to inhibit the ER (tamoxifen, other selective ER modulators, and the ER-degrader fulvestrant) or to suppress the production of estrogen in the body through ovarian ablation or suppression in premenopausal women, as well as aromatase inhibitors (AI) in postmenopausal women (Visovsky, 2014). Hormonal therapies have a long history of efficacy, reducing rates of recurrence in early-stage breast cancer and rates of progression in advanced disease, as well as improving overall survival (OS) in all stages of breast cancer (Early Breast Cancer Trialists’ Collaborative Group, 2005; Jones & Buzdar, 2004). They are also much better tolerated than most other treatment options. Thus, for most women with early-stage, hormone receptor–positive (HR-positive) disease, HTs are the preferred treatment option after surgery and radiotherapy (if needed). In some women with high-risk tumors, cytotoxic chemotherapy may be added to the treatment regimen. Similarly, HTs are preferred as initial therapy in most women with advanced and metastatic, HR-positive disease (National Comprehensive Cancer Network, 2017; Rugo et al., 2016).

Despite the efficacy of HT, many patients still experience recurrence or progression of their breast cancer during or after treatment. For example, the disease is resistant to HT in about half of patients, and eventually progresses in essentially all patients with ER-positive advanced (stage IV) breast cancer (Iwase & Yamamoto, 2015; Osborne & Schiff, 2011). When the disease progresses on one class of HT, it may respond to another class (Rugo et al., 2016), but those ensuing responses are often less durable with successive lines of therapy (Osborne & Schiff, 2011). Researchers studying the mechanisms of resistance to HT have found signaling pathways that can stimulate cancer cell growth when estrogen signaling is inhibited (Osborne & Schiff, 2011). Drugs targeting those alternative pathways have recently become available for use in combination with hormonal agents in appropriate patients with advanced, HR-positive breast cancer. In addition, two HTs with different mechanisms of action may be used concurrently in selected situations.

The combination of HT with targeted agents increases the burden of side effects in comparison to HT alone. As these combinations become widely used, advanced oncology practitioners will need to manage patients receiving even more complex treatment regimens. The successful use of these therapies is important not only for their benefits on disease progression, but also because they may prevent or delay the need for chemotherapy, which can introduce severe detrimental effects on quality of life (QOL). Indeed, the concept of chemotherapy-free survival is becoming an important goal of breast cancer therapy (Glück, Arteaga, & Osborne, 2011), as is maintenance of QOL. Beyond the challenges and side effects of treatment, patients with metastatic breast cancer require the support of skilled practitioners to help them manage the side effects of the disease and previous treatments (such as surgery and radiation), comorbidities, and the psychological and social impact of the disease.

## BIOLOGIC RATIONALE FOR COMBINATION THERAPIES

For treatment of ER-positive advanced breast cancer, two broad types of combination therapies are in use: (1) combinations of two HTs; (2) combinations of one HT with a targeted drug. The first type—combinations of two HTs—consists of the selective ER-degrader fulvestrant used in combination with an AI. Preclinical studies suggested that this combination may overcome some of the limitations of either type of agent used alone—such as resistance and duration of response (Jelovac, Macedo, Goloubeva, Handratta, & Brodie, 2005; Macedo, Sabnis, Goloubeva, & Brodie, 2008). As with AI monotherapy, this combination is appropriate only in postmenopausal women.

The ER participates in other cell signaling pathways such as the epidermal growth factor (EGF), human epidermal growth factor receptor 2 (HER2), and mammalian target of rapamycin (mTOR) pathways. The "crosstalk" between these pathways is thought to contribute to mechanisms of resistance to HT. The ability of fulvestrant to degrade the ER makes it less available to these other pathways, diminishing some of the molecular routes by which cancer cells can become resistant to hormonal agents (Osborne & Schiff, 2011; Osborne, Wakeling, & Nicholson, 2004). Those other pathways have also become important targets for the development of new classes of targeted therapies for breast cancer, some of which are associated with better outcomes when used in combination with hormonal agents.

One targeted therapy that has been approved for use in combination with hormonal therapy is palbociclib (Ibrance; Pfizer Inc., 2017). Palbociclib is an orally administered inhibitor of cyclin-dependent kinases 4 and 6 (CDK4/6), enzymes that promote entry into the cell cycle and become dysregulated in many cancers (Asghar, Witkiewicz, Turner, & Knudsen, 2015). In ER-positive breast cancer cells, several signaling pathways promote the activation of CDK4/6, which drives cell proliferation. By selectively inhibiting CDK4/6, palbociclib blocks the phosphorylation of downstream proteins essential for entry into the cell cycle (Asghar et al., 2015). In cultured, ER-positive, breast cancer cell lines, the combination of palbociclib and a hormonal agent demonstrated enhanced tumor cell sensitivity, and greater inhibition of proliferation than HT alone (Finn et al., 2009). Furthermore, palbociclib retained at least some activity in cells that had developed resistance to ER inhibitors (Finn et al., 2009; Thangavel et al., 2011).

Two other signaling pathways relevant to combination therapies in ER-positive breast cancer are the mTOR pathway and the HER2 pathway. Signaling through the phosphatidylinositol 3-kinase (PI3K)-Akt-mTOR pathway can phosphorylate the ER and promote estrogen-independent growth of breast cancer cells (Osborne & Schiff, 2011). The HER2 pathway interacts with the ER pathway in ways that can promote resistance to HT and estrogen-independent growth of breast cancer cells (Buzdar, 2009). These interactions have led to clinical studies of HT in combination with either mTOR inhibitors (such as everolimus [Afinitor]) or HER2 inhibitors (such as trastuzumab [Herceptin] and the HER1/HER2 inhibitor lapatinib [Tykerb]).

## CLINICAL STUDIES IN PATIENTS WITHOUT PRIOR HORMONAL THERAPY FOR RECURRENT OR METASTATIC DISEASE

Two studies assessed the combination of fulvestrant with the AI anastrozole in women with metastatic or relapsed breast cancer who had received no prior therapy for recurrent or metastatic disease, although many had received HT for early-stage disease.

The SWOG S0226 trial (Mehta et al., 2012) compared anastrozole alone to anastrozole plus fulvestrant, and about 40% of the patients had received tamoxifen for early-stage disease. In this trial, fulvestrant was administered as an initial loading dose of 500 mg followed by 250 mg at days 14 and 28, and every 28 days thereafter. The protocol was amended during the study to allow patients to receive 500 mg after this dose was shown to be superior to 250 mg in the CONFIRM trial (Di Leo et al., 2010). Patients who received combination therapy had longer median progression-free survival (PFS; 15.0 months; 95% confidence interval [CI] = 13.2–18.4) than those who received anastrozole alone (13.5 months; 95% CI = 12.1–15.1; hazard ratio [HR], 0.80; 95% CI = 0.68–0.94; *p* = .007), and longer median OS (47.7 vs. 41.3 months; HR, 0.81; 95% CI = 0.65–1.00; *p* = .05). It should be noted that 41% of the patients in the anastrozole arm switched to fulvestrant after progression on anastrozole, which would be expected to reduce the difference in treatment outcomes between the two arms.

The FACT trial (Bergh et al., 2012) enrolled women who had breast cancer recurrence at first relapse after primary treatment of localized disease, and about two-thirds had received endocrine therapy for early-stage disease. The treatment groups and regimens were similar to those in the SWOG 0226 trial, although monthly doses of fulvestrant (after the loading doses) remained at 250 mg. There were no significant differences between the two groups in terms of median time to tumor progression or OS. Although the trials had mixed results, the combination of a nonsteroidal AI and fulvestrant is currently a treatment option for first-line therapy of advanced, HR-positive breast cancer in women who had no prior adjuvant therapy or had relapsed at least 12 months after stopping adjuvant HT (Rugo et al., 2016). As always, an AI should be used only in postmenopausal women or in those whose ovarian function has been suppressed.

The combination of the AI letrozole and the CDK4/6 inhibitor palbociclib was studied in the phase II PALOMA-1 trial, which enrolled postmenopausal women with advanced ER-positive, HER2-negative breast cancer (Finn et al., 2015). Patients had received no systemic therapies for advanced disease, although about one-third had received prior HT for early-stage disease. In this first-line setting, the combination of letrozole and palbociclib was associated with significantly longer PFS than the standard treatment of letrozole monotherapy, and the combination was provisionally approved on the basis of that trial. The phase III PALOMA-2 trial was conducted in a similar patient population to confirm the results of the phase II study. Women in the letrozole plus palbociclib combination therapy group had a median PFS of 24.8 vs. 14.5 months for women in the letrozole-alone group (HR, 0.58; 95% CI = 0.46–0.762; *p* < .001; Finn et al., 2016).

Ribociclib (Kisqali) is the second CDK4/6 inhibitor to be approved in combination with an AI as the first HT for postmenopausal women with HR-positive, HER2-negative advanced or metastatic breast cancer. The approval was based on a preplanned interim analysis of the data from the MONALEESA-2 trial. After 18 months of treatment, 63.0% (95% CI = 54.6–70.3) of women in the combination therapy group met the criteria for PFS compared with 42.2% (95% CI = 34.8–49.5) in the letrozole monotherapy group (HR, 0.56; 95% CI = 0.43–0.72; *p* < .001). The median PFS was not reached for the combination (95% CI = 19.3–not reached) but was 14.7 months for letrozole monotherapy (95% CI = 13.0–16.5; Hortobagyi et al., 2016).

Both ribociclib and palbociclib have been granted FDA approval for coadministration with any AI (Novartis Pharmaceuticals, 2017; Pfizer Inc., 2017). The approval of palbociclib was based on the results from the PALOMA-1 (Finn et al., 2015) and PALOMA-2 (Finn et al., 2016) studies. The positive results from PALOMA-2 and MONALEESA-2 indicate that the combination of letrozole and a CDK4/6 inhibitor is likely to become a major treatment option for first-line therapy of advanced, ER-positive, HER2-negative breast cancer in postmenopausal women.

A subset of patients with HR-positive advanced disease also have tumors that overexpress HER2 (HER2-positive). Three trials explored combinations of a nonsteroidal AI (letrozole or anastrozole) with a HER2 antagonist (lapatinib or trastuzumab) in such patients. One trial also included HER2-negative patients and confirmed that the addition of lapatinib was of no benefit in those patients (Johnston et al., 2009). However, in HER2-positive patients, the addition of lapatinib to letrozole significantly prolonged PFS (8.2 vs. 3.0 months; HR for progression, 0.71; 95% CI = 0.53–0.96; Schwartzberg et al., 2010). Two other trials found that the addition of trastuzumab to anastrozole (Kaufman et al., 2009) or letrozole (Huober et al., 2012) improved PFS compared with AI monotherapy. A trial of lapatinib added to fulvestrant in this setting found no benefit (Burstein et al., 2014). Currently, combination therapy with an AI and a HER2-targeted agent is not a standard approach in patients with ER-positive, HER2-positive disease because it has not shown a survival advantage (Rugo et al., 2016). Nevertheless, patients with HER2-positive disease can still be candidates for HT, especially those with more indolent disease.

## CLINICAL STUDIES IN PATIENTS WHO PROGRESSED ON PRIOR HORMONAL THERAPY FOR ADVANCED BREAST CANCER

Disease progression occurs when target lesions grow larger over time and is measured using RECIST guidelines (Eisenhauer et al., 2009). Combinations of two HTs are not recommended in patients whose advanced breast cancer had progressed on prior HT (Rugo et al., 2016). Instead, the combination of a hormonal agent with a targeted agent may be more promising in this setting. The phase III PALOMA-3 study (Turner et al., 2015) enrolled women (pre- or postmenopausal) with ER-positive, HER2-negative advanced breast cancer that had relapsed or progressed on prior HT (about 25% had received prior HT only for early-stage disease). The group receiving combination therapy (fulvestrant and palbociclib) had significantly longer PFS (9.2 months; 95% CI = 7.5–not reached) than the group receiving fulvestrant monotherapy (3.8 months; 95% CI = 3.5–5.5 months; HR, 0.42; 95% CI = 0.32–0.56; *p* < .001; Turner et al., 2015). It should be noted that in comparison, PALOMA-2 was conducted in postmenopausal, ER-positive, HER2-negative breast cancer, with no prior treatment for advanced disease (Finn et al., 2016).

The combination of an mTOR inhibitor (everolimus) with exemestane has also been found to be more effective in second-line therapy than exemestane monotherapy. The phase III BOLERO-2 study (Baselga et al., 2012) enrolled women with advanced breast cancer who had recurrence or progression of disease on a nonsteroidal AI. Many of the patients had also received prior therapy with tamoxifen (48%) or fulvestrant (16%). The group that received the combination of everolimus and exemestane had a PFS of 11.0 months compared with 4.1 months in the exemestane monotherapy group (HR, 0.38; 95% CI = 0.31–0.48; *p* < .0001; Yardley et al., 2013). Patients in this study could be considered a mix of first- and second-line patients; therefore, the study supports the combination of an AI and everolimus in either setting. However, a study of a different mTOR inhibitor, temsirolimus (Torisel), added to letrozole for first-line therapy found no evidence of benefit compared with letrozole alone (Wolff et al., 2013).

Combining abemaciclib (Verzenio), a selective CDK4/6 inhibitor, with fulvestrant significantly improved PFS and objective response rate (ORR) compared with fulvestrant monotherapy in women with HR-positive, HER2-negative advanced breast cancer who progressed while receiving either first-line, neoadjuvant, or adjuvant endocrine therapy. In the MONARCH 2 phase III study, women with any menopausal status and progressive disease who received no chemotherapy treatment and up to one prior endocrine therapy were eligible. Patients treated with the fulvestrant and abemaciclib combination achieved a median PFS of 16.4 months compared with 9.3 months in the fulvestrant monotherapy arm (HR, 0.553; 95% CI = 0.449–0.861; *p* < .001) and an ORR of 35.2% vs. 16.1%, respectively (*p* < .001; Sledge et al., 2017). The addition of abemaciclib to fulvestrant resulted in a complete response (CR) in 14 patients compared with 1 CR in the fulvestrant control group (Sledge et al., 2017). The ORR noted in MONARCH 2 is the highest observed in a phase III study conducted with patients whose disease progressed while receiving prior endocrine therapy.

These results prompted the FDA approval of abemaciclib (starting dose: 150 mg twice daily) and fulvestrant combination therapy for women with HR-positive, HER2-negative advanced or metastatic breast cancer with disease progression following endocrine therapy. Abemaciclib is also approved as a monotherapy (starting dose: 200 mg twice daily) for the treatment of adult patients with HR-positive, HER2-negative advanced or metastatic breast cancer with disease progression following endocrine therapy and prior chemotherapy in the metastatic setting (Eli Lilly USA, 2017).

## IMPLICATIONS FOR THE ADVANCED PRACTITIONER

In clinical studies evaluating therapies for advanced breast cancer, the outcomes that receive the most attention are typically PFS, ORR, time to progression, and OS. These outcomes are important for reducing tumor and metastatic burden, which can help maintain QOL in patients with metastatic disease (Gradishar, 2014). However, treatment side effects can also decrease QOL, as can disease- and treatment-related psychological issues including anxiety and depression (Gradishar, 2014; O’Shaughnessy, 2014). Individual patients have different views about how they weigh the duration of life vs. QOL. Furthermore, those views may change at different stages of disease, and they may be influenced by external factors such as family responsibilities and social support. Recognizing the importance of QOL, major organizations now recommend that palliative care for patients with cancer begin at the time metastatic disease is diagnosed, involve multidisciplinary teams, and be an integral part of treatment (Ferrell et al., 2017). The essential components of this integrated palliative care approach are listed in Table 1. This approach can improve patient outcomes and QOL, reduce depression, and increase the satisfaction with care (Ferrell et al., 2017).

**Table 1:**
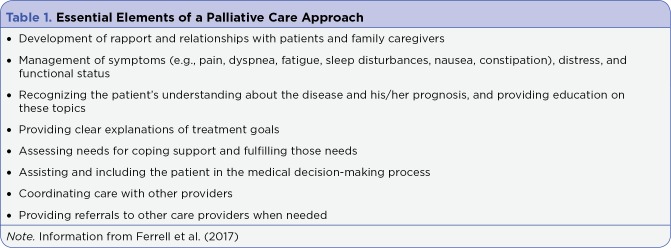
Essential Elements of a Palliative Care Approach

Whenever possible, practitioners should encourage and support patients to exercise an appropriate degree of health-care autonomy, so that they are directly and openly involved in treatment planning, and take part in informed decision-making with their practitioner. In this way, patients can express their goals, desires, and concerns, which can be factored into the treatment plan along with clinical goals. Such participation and "buy-in" from the patient is also essential for optimal adherence to the chosen treatment, especially since many of the combination therapies are oral agents that patients manage independently between clinic visits. Other factors that may influence adherence are side effects, the complexity of the dosing regimen, and the method of drug administration. An appropriate level of patient autonomy and participation in treatment planning requires patient education from practitioners who can provide patients with reliable information about treatment options and expected side effects. When patients are knowledgeable about these issues, it can improve treatment adherence and tolerability.

**Figure 1:**
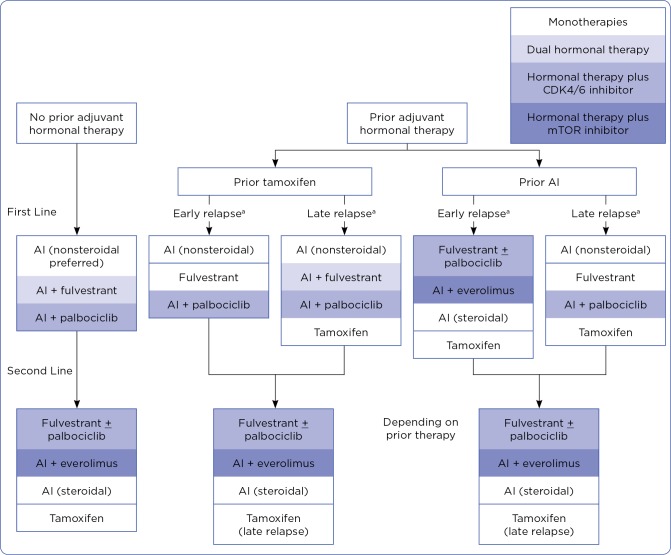
Treatment options for hormonal therapy of advanced, hormone receptor–positive breast cancer in postmenopausal women. Modified from American Society of Clinical Oncology Guidelines (Rugo et al., 2016). As of January 2016, the combination therapies approved to treat advanced breast cancer in the United States are: letrozole + palbociclib, fulvestrant + palbociclib, and exemestane + everolimus. AI = aromatase inhibitor. a Early relapse, ≤ 12 months after adjuvant hormonal therapy; late relapse, > 12 months after adjuvant hormonal therapy.

Treatment selection can be tailored to the needs and wishes of the patient, taking into consideration their individual history and tolerability of previous regimens. Treatment guidelines are intended to aid in the selection of therapy alongside the judgment of the clinical team and the patient’s goals and desires. Figure 1 summarizes current American Society of Clinical Oncology (ASCO) guidelines for HT in postmenopausal women with advanced, HR-positive breast cancer (Rugo et al., 2016). Hormonal monotherapy, dual HT (an AI plus fulvestrant), and combinations of HT with targeted therapies have prominent roles in the treatment of both pre- and postmenopausal women. However, ovarian suppression would be needed for premenopausal women. After the ASCO guidelines were released, results of the PALOMA-2 (Finn et al., 2016) and MONALEESA-2 (Hortobagyi et al., 2016) studies were published, showing strong evidence for improved outcomes in postmenopausal women who had not received prior treatment for advanced disease when a CDK4/6 inhibitor is added to letrozole (Finn et al., 2016). Therefore, future guidelines may add these combination therapies as an option for those patients.

The addition of oral targeted agents, such as palbociclib or everolimus, to HT is associated with a substantial increase in the frequency and severity of side effects. Some of the most common side effects are described in Table 2, but practitioners should review with each patient all potential side effects for any treatment and approaches to management. Patients also need a clear understanding about when to contact a member of the care team for help in managing treatment and its side effects. Maintaining those open lines of contact is essential, since attempts by patients to endure or wait out side effects may have severe consequences. When patients can immediately discuss side effects and other concerns with members of the care team, the safety and effectiveness of oral therapies are enhanced.

**Table 2:**
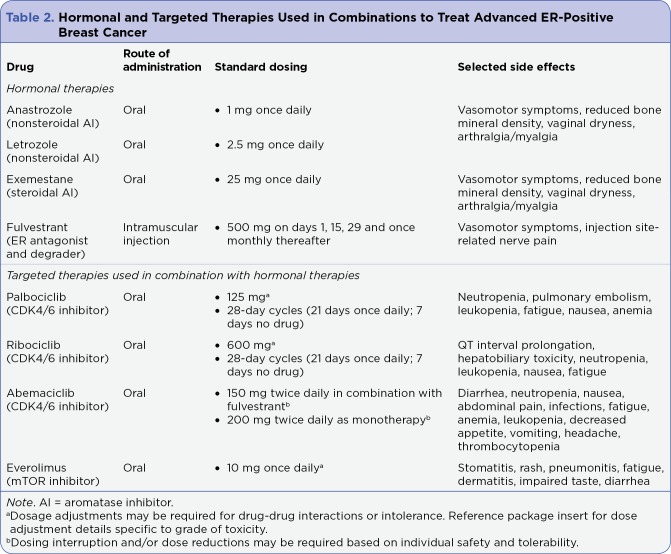
Hormonal and Targeted Therapies Used in Combinations to Treat Advanced ER-Positive Breast Cancer

When palbociclib was added to HT in clinical trials, it was associated with distinct side effects not usually seen with HT alone. In the PALOMA-2 trial, for example, grade 3/4 hematologic toxicities were common in the palbociclib plus letrozole arm. Neutropenia (66% vs. 1%), leukopenia (25% vs. 0%), anemia (5% vs. 2%), and thrombocytopenia (2% vs. 0%) were all more common in the combination therapy group than the letrozole monotherapy group (Finn et al., 2016). Similar side effects were observed when palbociclib was used in combination with fulvestrant (Turner et al., 2015). Hematologic values may be even less stable in patients who have had prior chemotherapy.

Patients receiving palbociclib must understand the need for monitoring of blood counts, which should occur before starting therapy, at the beginning of each dosing cycle, and at day 15 of the first 2 cycles (Pfizer Inc., 2017). For example, one patient was prescribed palbociclib at 125 mg and letrozole at 2.5 mg for newly diagnosed stage IV ER-positive/HER2-negative breast cancer that had metastasized to the bone. Her complete blood count (CBC) was normal on days 1 and 14 of cycle 1; however, on day 1 of cycle 2, her neutrophil count was 0.9 × 10³/μL, a grade 3 toxicity. She was afebrile and the remainder of her CBC was normal. It was decided to hold palbociclib for 1 week in accordance with the prescribing information (Pfizer Inc., 2017), delaying initiation of cycle 2; however, during this week the patient continued to receive letrozole once daily. A CBC 1 week later revealed a neutrophil count of 1.5 × 10³/μL, a grade 1 toxicity. Palbociclib was restarted at the same initial dose per the prescribing information (Pfizer Inc., 2017). Her CBC on day 14 was normal, as well as on day 1 of cycle 3. The patient continued taking the full dose of palbociclib for 26 months until time of disease progression.

Palbociclib is administered in 28-day dosing cycles (21 days on, 7 days off), which can lead to confusion for patients who are also concurrently taking HT such as letrozole (daily) or fulvestrant (monthly). A useful solution is a 28-day pill box, which can also help patients keep track of the dosing cycle and when blood counts are needed. Patients should be advised to take palbociclib with food, to avoid grapefruit juice, and to report any medications or changes in medications (including herbs or supplements) because drug-drug interactions are common (Pfizer Inc., 2017). Prescribers and practitioners should consult with the patient’s pharmacy about other medications the patient is taking, especially CYP3A inducers and inhibitors, as these interactions are commonly seen with palbociclib and everolimus. Inadequate understanding of drug-drug interactions may lead to ineffective therapy or unnecessary and possibly dangerous side effects resulting from alterations in drug metabolism. For patients who experience hematologic or nonhematologic toxicities with palbociclib, dosing modifications are described in the prescribing information (Pfizer Inc., 2017).

Ribociclib has a dosing schedule and hematologic side effect profile similar to those for palbociclib. The most frequent grade 3/4 hematologic side effects from the MONALEESA-2 study for the letrozole plus ribociclib combination vs. letrozole monotherapy were neutropenia (74.3% vs. 5.2%), leukopenia (21.0% vs. 0.6%), and lymphopenia (6.9% vs. 0.9%). An increase in QT interval occurred in 9 patients (2.7%), but most of these resolved without interruption of treatment. Electrocardiogram testing on days 1 and 14 of cycle 1 and day 1 of cycle 2 are recommended, and concomitant use with other drugs that may prolong QTc intervals are discouraged. In addition, monitoring of electrolytes at the beginning of every cycle is recommended.

As ribociclib is a strong CYP3A4 inhibitor, dose adjustments may be required if it is being taken concomitantly with another drug metabolized through this pathway. Two liver enzymes also increased: alanine aminotransferase (9.3% vs. 1.2%) and aspartate aminotransferase (5.6% vs. 1.2%; Hortobagyi et al., 2016). As such, liver enzymes should be evaluated on days 1 and 14 of the first two cycles and day 1 of subsequent cycles. Treatment with ribociclib may require interruption, reduction, or discontinuation if liver function tests suggest unacceptable toxicities (Novartis Pharmaceuticals, 2017). Ribociclib is provided as 200 mg tablets with a starting dose of 600 mg/day taken with or without food once daily for 21 days followed by 7 days off treatment. Additional dose reductions are suggested as 400 mg daily and 200 mg daily, respectively, should the patient experience side effects that require a reduction. The prescribing information can be consulted for additional information and patient guidance (Novartis Pharmaceuticals, 2017).

The addition of everolimus to exemestane was also associated with several distinct side effects. Stomatitis (any grade) was observed in 59% of patients receiving the combination, compared with 12% receiving exemestane alone (grade 3/4, 8% vs. 0%; Yardley et al., 2013). An approach for reducing or preventing stomatitis is the use of 10 mL dexamethasone mouth rinse (0.5 mg/5 mL), 4 times daily, starting at the onset of everolimus use. The SWISH trial showed that such a mouth rinse, used prophylactically, reduced rates of stomatitis (all grades) in patients receiving everolimus and exemestane (Rugo et al., 2017). The authors suggested that this approach should be a standard of care for such patients. In addition, other mouth rinses and toothpastes should be alcohol-free to minimize xerostomia and other oral adverse effects of therapy.

Another complication associated with everolimus is pneumonitis. Often it is slow to develop and symptoms may present between office visits. Thus, the care team should have a low threshold for ordering computed tomography (CT) imaging in patients receiving everolimus who report shortness of breath, activity intolerance, or chronic coughing; a chest x-ray is not sensitive enough to detect this complication. Pneumonitis can be managed by a reduction in dose (approximately 50%), interruption or discontinuation of therapy, and the use of corticosteroids may be considered until symptoms improve to grade 1 or less (Novartis Pharmaceuticals, 2016). For example, one patient began treatment with exemestane (25 mg/day) plus everolimus (10 mg/day) after she developed stage IV breast cancer that had metastasized to the bone and lung while receiving adjuvant nonsteroidal endocrine therapy. The patient was doing well, with normal laboratory studies at the time of her 3-month reevaluation, and imaging studies showed response in the bone and lung with no new concerning findings. As such, treatment was continued at the same dose. However, at 6 months, while her disease continued to respond, the patient noted that she was becoming progressively short of breath while performing day-to-day activities, suggesting a grade 2 lung toxicity. A chest CT scan showed new bilateral subpleural opacities suspicious for drug-related inflammation, specifically pneumonitis. Everolimus was held, exemestane was continued, and systemic steroids were initiated until symptoms resolved, returning to baseline status 3 weeks later. Everolimus was restarted at 5 mg/day with no return of symptoms, and her disease continued to respond while on a reduced dose of everolimus for more than 2 years.

Patients should be educated to take everolimus at a consistent time each day with a full glass of water, with or without food. Review each patient’s medication list during each visit and empower them to notify a member of the health-care team before starting or changing any medications between visits, because of the potential for drug-drug interactions. Finally, individuals with lower performance status may need to start at a low dose of everolimus and gradually titrate toward the full dose only as tolerated; such titration may help to avoid potentially serious side effects that are often slow to reverse.

The addition of abemaciclib to fulvestrant caused more diarrhea, neutropenia, nausea, fatigue, and abdominal pain than fulvestrant monotherapy in the MONARCH 2 study, but most of these occurred at a grade 1 or 2 severity (Sledge et al., 2017). Six patients in the abemaciclib and fulvestrant arm also developed febrile neutropenia. Another important adverse event to note is a higher incidence of infections in the abemaciclib and fulvestrant arm (42.6%) than in the fulvestrant monotherapy arm (24.7%) regardless of relatedness. These infections were predominately of grade 1 to 2 severity (6.6% in the abemaciclib arm vs. 3.6% in the fulvestrant monotherapy arm were grade ≥ 3; Sledge et al., 2017). Three patients on abemaciclib within MONARCH 2 trials died while on study, two from sepsis, both of whom did not have appropriate dose reductions or follow guidance regarding granulocyte colony stimulating factor administration (Sledge et al., 2017). Thromboembolic events were the most commonly reported serious adverse event reported in the combination therapy arm compared with the fulvestrant monotherapy arm (2.0% vs. 0.4%, respectively; Sledge et al., 2017). Avoid coadministration of abemaciclib and strong CYP3A4 inhibitors, as this leads to increased exposure of abemaciclib and may lead to increased toxicity (Eli Lilly USA, 2017). Strong CYP3A inducers may lead to decreased plasma concentration of abemaciclib and consequently reduced activity (Eli Lilly USA, 2017). Similar to other CDK4/6 inhibitors, coadministration of abemaciclib with any other CYP3A4 inducer or inhibitor should be closely monitored, and avoided if deemed safe.

Similar to the clinical studies leading to the implementation of palbociclib and ribociclib, the findings from the MONARCH 1 and MONARCH 2 studies have resulted in monitoring recommendations. It is important to note that depending on the setting in which abemaciclib is being initiated, the dose will vary. When administered in conjunction with fulvestrant, the starting dose of abemaciclib is 150 mg twice daily; however, when administered as monotherapy, the starting dose is 200 mg twice daily (Eli Lilly USA, 2017). Current guidelines for monitoring include evaluation of CBC, aspartate aminotransferase, alanine aminotransferase, and serum bilirubin every 2 weeks for the first 2 cycles, followed by monthly for the following 2 cycles. Thereafter, laboratory monitoring is at the discretion of the ordering provider (Eli Lilly USA, 2017). Guideline-based dose reductions for toxicities, including hematologic derangements, hepatotoxicity, and diarrhea, can be accessed within the prescribing information (Eli Lilly USA, 2017).

## CONCLUSION

Combination therapies for advanced breast cancer—involving dual hormonal agents or hormonal agents combined with targeted therapies—are extending the lives of patients in a manner that can often also provide an acceptable QOL. Nevertheless, many patients need ongoing support beyond that related to treatment timing and scheduling—such as prevention and management of complications and side effects, avoidance of drug-drug interactions, access to providers of physical and psychosocial support, and knowledge about how and when to contact members of the care team. With recent and ongoing advances in the treatment of breast cancer, and the use of more complex treatment regimens such as combination HT, it is more important than ever to use a team approach for patient care, with the patients themselves included within the team. Patients also need complete and consistent education about self-management of the disease, its treatment, and side effects. The advanced practitioner has an essential role in helping patients understand and adapt to their diagnosis and prognosis, as well as helping them feel successful with treatment. Fortunately, survival and QOL of patients with metastatic breast cancer continues to improve, allowing many more men and women with the disease to measure their remaining lives in quality-filled years. Thankfully, these improvements are allowing many patients to shift their oncologic focus from dying from the disease to learning how to live with it instead.

**Acknowledgment**

Writing assistance by Ken Scholz, PhD, and editorial support were provided by The Lockwood Group, Stamford, CT, and funded by AstraZeneca LP.
